# A Decade of Drug-Eluting Technology in Peripheral Arterial Disease: Blurred by Dissembling Evidence

**DOI:** 10.1007/s00270-016-1476-1

**Published:** 2016-09-26

**Authors:** J. A. Reekers, C. J. M. de Vries

**Affiliations:** 1 Department of Radiology, AMC, University of Amsterdam, Meibergdreef 9, 1105 AZ Amsterdam, The Netherlands; 2 Department of Medical Cell Biochemistry, AMC, University of Amsterdam, Meibergdreef 9, 1105 AZ Amsterdam, The Netherlands

## Introduction

In 2005 the results of the Sirocco II trial, comparing the safety and efficacy of the sirolimus-eluting stents and bare nitinol stents in the superficial femoral artery were published [1]. In this study, finally, no statistically significant differences in any of the outcomes could be shown. Since then there has been a complete revolution in drug-eluting technologies for peripheral vessels. There have been fierce debates about coatings, types of drugs, release profiles, and doses. The introduction of the drug-eluting balloons provoked a new debate about not leaving something behind. Unfortunately, the majority of the literature is about these technical details and safety and there are only a few randomized trials available for clinical analysis. Although the results of all these new devices have been presented as very successful or at least very promising, there were also a few critical voices at the background [2]. These voices however have not had much attention, moreover they were seen as silly twaddle or flawed arguments.

## How Does Drug-Eluting Technology Work on the Vessel wall?

Both balloon expansion and stent placement provoke severe damage to the vascular endothelium. This single cell layer needs to be repaired to avoid local thrombotic events and once the endothelium is recovered, this also supports quiescence of the underlying smooth muscle cells. Healthy and functional endothelium is crucial to provide an anti-coagulant surface and it delimits smooth muscle cell growth and thus neointimal lesion formation [3]. The ideal drug for local delivery should inhibit the proliferation of smooth muscle cells and simultaneously promote endothelial cell growth.

The most frequently used drugs for local vascular application by balloons and stents are the cytostatic drug paclitaxel and the immunosuppressive medicine sirolimus and its derivatives [4]. Paclitaxel enters the cell to bind and stabilize tubulin polymers thereby disturbing regular metaphase spindle formation during cell division. As a result, chromosomes cannot segregate and cell division is blocked and this is often followed by cell apoptosis, making paclitaxel an effective drug in several cancer therapies. Initially sirolimus, also known as rapamycin, was shown to block the activation of T and B cells through inhibition of the so-called mechanistic Target of Rapamycin (mTOR) intracellular signaling pathway. Later on, it became clear that inhibition of mTOR also compromises the proliferation of many different cell types. Since then, multiple sirolimus analogues have been developed such as everolimus, tacrolimus, and biolimus to treat specific types of cancer. Paclitaxel and limus derivatives inhibit the proliferation of smooth muscle cells very efficiently, explaining their un-surpassed effectiveness in preventing intimal hyperplasia. The mere fact that these drugs also ameliorate the recovery of the endothelial cell layer after intervention, indicates that there still is a need for more cell-type-specific drugs in drug-eluting technology to improve safety and reduce thrombosis risk. Given that local balloon-mediated delivery involves application of a single dose of drug in the vessel wall and that stents release drugs for a period of only 2–4 weeks, it is remarkable that even after 12 months there is limited intimal hyperplasia and incomplete endothelialization of the treated vessel segment [3]. This could point in the direction of permanent vessel wall damage with even possible future implications. There are some new technologies underway that potentially will work more targeted, not blocking the endothelial cell repair, but further clinical investigations need to be done first [5, 6].

## Where Do We Stand?

By now there is enough good evidence to show that drug-eluting technology does have an impact on the vessel wall and intimal hyperplasia. Two high-quality systematic reviews show improved primary vessel patency (high-quality evidence), binary restenosis rate (moderate quality evidence), and target lesion revascularization (low-quality evidence) for up to 12 months [2, 7]. However, for real endpoints that matters to the patients’ health and wellbeing like improved walking distance, Quality of life, amputation free survival, death, or change in Rutherford category during 12 months follow-up no improvement could be shown in these two meta-analyses. A swift interpretation of these data could lead to the conclusion that better patency does not translate into better outcome and that drug-eluting technology is yet another endovascular myth.

But is this really true or are we to early with our judgement? One of the main flaws in almost all studies on drug-eluting technology are the inclusion criteria and study endpoints. If one includes two completely different types of patients, one group with claudication and one group with critical limb ischemia (CLI) in a randomized trial and then applies the same endpoints, like amputation or improved walking distance, there will never be any difference. 95 % of the patients with claudication are never at risk for amputation and CLI patients cannot be measured for improved walking distance. This is also confirmed by another high-quality meta-analysis showing statistically significant superiority of drug-eluting stents over bare metal stents for late lumen loss and TLR, but again with no benefit in amputation or mortality [8].

The LEVANT trial comparing drug-eluting ballooning (DEB) with percutaneous transluminal angioplasty and stent on indication, in the superficial femoral artery with a 2:1 randomization, included 148 patients with mild claudication, 290 patients with severe claudication, and 38 patients with ischemic rest pain. There were no patients with tissue loss included (Rutherford 5 and 6) [9]. No difference in meaningful clinical endpoints between both groups was seen [9]. We know that patients with mild claudication do fine without any intervention and that in patients with critical limb ischemia outcome is not always directly related to infrapopliteal vessel disease or ABI. Also not every patient with rest pain is at high risk for amputation. This study is therefore flawed because of the wrong inclusion and the wrong endpoints for this targeted group, and therefore this study was unable to show clinical benefit.

In the PADI study, which is a randomized trial to compare DES to PTA with or without bare stent, only patients with critical limb ischemia (Rutherford category ≥4) and infrapopliteal lesions were included [10]. The major amputation rate remained lower in the DES group until 2 years post-treatment, with a trend toward significance (*P* = 0.066). The fact that it did not reach significance could be because of the small sample size, the use of a sub-optimal coronary stent in the DES group versus a none coronary stent in the PTA group, and the fact that no sub-analysis for the grades of ischemia was possible because of the small sample size.

The important lesson to learn is that included patients and endpoints must match to be able to obtain a meaningful result of any trial.

## Conclusion

The statement that drug-eluting technology gives better clinical outcome compared to standard pta and that this is a proven technology is not supported by the current literature. However, the statement that drug-eluting technology does not work is also incorrect, moreover there is enough evidence that shows the inhibiting effects on the repair of vessel wall cells after pta. The evidence that drug-eluting techniques have a positive effect on the inhibition of intimal hyperplasia still not translates into a better clinical outcome. TLR, VLR, and clinically driven re-intervention are proxy endpoints and a major concern regarding its questionable relevance and deceiving nature [11]. We still just need good studies with proper patient selection and matching clinically relevant endpoints. The PADI study is an example of how such a study should be designed. Is this ever going to happen? The main stakeholders are satisfied with the current state of evidence, the majority of interventional radiologists and vascular surgeons believe in drug-eluting technology, as it became clear from the audience pooling at the 2016 Charing Cross meeting (London, UK). So there is no urgent need to start a new study as the goals for the industry and most physicians have been obtained. The alarming publicity coming from the IN.PACT DEEP trial showing a trend towards higher amputation in the DEB—arm will also not be a motivation for industry to support another trial like this [12]. Although this increase in amputation in the DEB—arm of the study could be explained by other external circumstances [13]. But without proper clinical evidence the main stakeholder in this discussion, the patient, is the one who finally pays the extra costs for this new technology without any proof of clinical efficacy. And that conclusion by itself should be a motivation for every interventionalist to get the evidence on the table as soon as possible.
